# Periostin as a Biomarker of the Amniotic Membrane

**DOI:** 10.1155/2012/987185

**Published:** 2012-07-01

**Authors:** Mariya P. Dobreva, Larissa Lhoest, Paulo N. G. Pereira, Lieve Umans, Susana M. Chuva de Sousa Lopes, An Zwijsen

**Affiliations:** ^1^Laboratory of Developmental Signaling, VIB11 Center for the Biology of Disease, VIB, 3000 Leuven, Belgium; ^2^Center for Human Genetics, KU Leuven, 3000 Leuven, Belgium; ^3^Laboratory of Molecular Biology (Celgen), Department of Development and Regeneration, KU Leuven, 3000 Leuven, Belgium; ^4^Department of Anatomy and Embryology, Leiden University Medical Centre, 2333 ZC Leiden, The Netherlands

## Abstract

Tracing the precise developmental origin of amnion and amnion-derived stem cells is still challenging and depends chiefly on analyzing powerful genetic model amniotes like mouse. Profound understanding of the fundamental differences in amnion development in both the disc-shaped primate and human embryo and the cup-shaped mouse embryo is pivotal in particular when sampling amniotic membrane from nonprimate species for isolating candidate amniotic stem cells. The availability of molecular marker genes that are specifically expressed in the amniotic membrane and not in other extraembryonic membranes would be instrumental to validate unequivocally the starting material under investigation. So far such amniotic markers have not been reported. We postulated that bone morphogenetic protein (BMP) target genes are putative amniotic membrane markers mainly because deficiency in one of several components of the BMP signaling cascade in mice has been documented to result in defective development of the early amnion. Comparative gene expression analysis of acknowledged target genes for BMP in different extraembryonic tissues, combined with *in situ* hybridization, identified *Periostin (Postn)* mRNA enrichment in amnion throughout gestation. In addition, we identify and propose a combination of markers as transcriptional signature for the different extraembryonic tissues in mouse.

## 1. Introduction

The amnion is the innermost extraembryonic membrane that surrounds the foetus of amniotes and delineates the fluid-filled amniotic cavity, thereby providing a confined environment within the conceptus and conferring protection and shock resistance. In most amniotes, the amnion is a thin and avascular transparent membrane. In recent years, human term amnion has attracted considerable attention because amniotic-ectoderm- and mesoderm-derived cells can differentiate into cells from the three germ layers in cell culture. Furthermore, resident stem cell-like cells in the amniotic ectoderm have been reported (reviewed in [1, 2], this issue). In addition, the low immunogenicity of the amnion makes this “medical waste” tissue of great interest for (regenerative) medicine. Indeed, the amnion has been used for over a century as a wound dressing.

Recently, studies aiming to explore the presence and origin of amniotic stem cells have been accomplished, using much more powerful genetic model organisms, such as, mouse and rat. Despite the fundamental differences in amnion development in the disc-shaped primate embryo and in the cup-shaped mouse embryos (for review: [2]), amniotic-membrane and amniotic-fluid derived cells with stem-cell-like features have been isolated from mouse and rat [3, 4].

**Figure 1:**
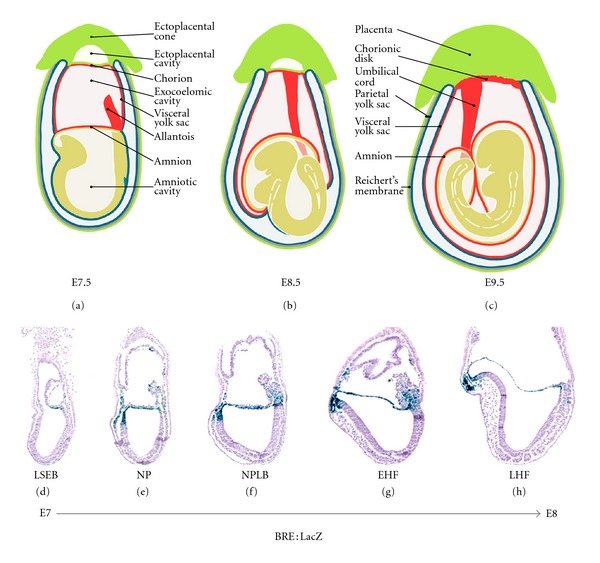
(a)–(c) Schematic representation of a mouse embryo illustrating the position of the extraembryonic tissues before and after axial rotation. During the process of axial rotation the embryo becomes enwrapped in its extraembryonic membranes. Extraembryonic mesoderm is shown in red; yellow represents amniotic ectoderm and embryonic ectoderm (embryonic mesoderm is not depicted); green represents trophectoderm-derived extraembryonic lineages; blue shows extraembryonic endoderm. For more detailed description of the extraembryonic membranes, see [2]. (d)–(h) Sagittal sections through BRE: LacZ mouse embryos in the time range between amnion closure (E7.0) and head fold stages (E8.0). X-gal staining (blue) for *β*-galactosidase detection in BRE : LacZ heterozygous embryos [5] reports dynamic SMAD1/5/8 mediated BMP signaling in the developing amnion. Abbreviations: E: embryonic day; LSEB: late streak-early bud; NP: neural plate; NPLB: neural plate-late bud; EHF: early head fold; LHF: late head fold.

In human, both amnion and chorion surround the embryo and both membranes fuse during the second trimester of pregnancy, while the yolk sac remains rudimentary [2]. In contrast, in mouse, the chorion will never fuse with the amniotic membrane after the physical separation of the amniochorionic fold shortly after gastrulation at embryonic day (E)7.0 [6]. The chorion becomes incorporated in the chorionic disk of the placenta, whilst the amnion becomes surrounded by the visceral yolk sac, except in the part of the chorionic disk. Importantly, amnion on the one hand and yolk sac and chorionic disk on the other hand remain spaced by the fluid-filled exocoelomic cavity (Figures 1(a)–1(c)). In mouse embryos, the amnion consists throughout gestation of a simple bilayered membrane of squamous mesoderm and ectoderm, which face the exocoelomic and amniotic cavity, respectively.

Awareness of the fundamental different surrounding tissues of human and mouse amnion is important when collecting amniotic membrane from nonprimate species to isolate the so-called amniotic stem cells [2]. The availability of molecular markers that are specifically present in amnion and not in the other extraembryonic membranes, or in the respective fluids, would therefore be helpful to characterize unequivocally the initial starting material from which stem cells are isolated.

Spatial gene expression studies rarely include information on expression in amnion, in part because this tissue appears prone to be neglected or discarded, but also because it is often hard to distinguish low expression levels from background staining due to the flat cellular architecture of this stretched membrane. To our knowledge, amnion-specific genes have not explicitly been reported, but the physiologic features of the amnion may hint towards putative candidate amnion markers. The amnion is a very elastic tissue that resists to increasing stretch. Hence, progressively, a basal lamina composed of collagen, laminin, nidogen, and fibronectin fibers forms between the amniotic ectoderm and mesoderm [7, 8]. The amniotic epithelium acquires an increasing number of microvilli at the surface, which may be associated with enhanced filtering and transport capacity across the membrane. Mouse models with impaired amnion development may also tip-off candidate amnion markers. Remarkably few mouse mutants displaying defects in amnion formation have been described (reviewed in [6]), in contrast to the many mutants with defects in allantois [9] or placenta [10]. Many of the mutants that display defects primarily related to amnion development seem to point at impaired bone morphogenetic protein (BMP) signaling (*Bmp2, Smad5*) or are found in genes that encode putative modulators of BMP signaling (*Amn, Bmp1*). Amnionless (*Amn*) mutants develop the most specific defects because they lack an amnion, whereas chorion, yolk sac blood islands, and allantois develop normally [11]. *Bmp2* null embryos fail to close the amnion [12]. Mice deficient in *Smad5*, an intracellular mediator of BMP signaling, also show delayed amnion closure, in addition to local amnion thickenings that contain ectopic stem cell-like cells, haematopoietic and endothelial cells [13–15]. Several ligands may elicit BMP signaling in amnion. *Bmp4* is expressed abundantly in mouse amnion, but *Bmp2 *and *Bmp7 *transcripts have also been reported in amnion and adjacent tissues [16–18].

The crucial role for BMP signaling in amnion development made us hypothesize that target genes for BMP are candidate markers of the amniotic membrane. Given the poor documentation of the expression of such target genes in the developing amnion and its neighboring tissues, we have performed a comparative gene expression analysis of several such target genes in different extraembryonic tissues. Transcripts for periostin (*Postn*) appeared to be highly enriched in mouse and human amnion at different stages of development. Periostin is a secreted ECM protein that can interact with different ECM proteins and integrins and that is induced by transforming growth factor (TGF)*β* and BMPs in tissues undergoing remodeling or active stress (reviewed in [19]). *In situ* hybridization analysis confirmed the amnion-enriched localization of *Periostin* mRNA in amnion. We propose to use a combination of *Periostin* and *AP-2* as biomarkers for developing mouse amniotic membrane.

## 2. Materials and Methods

### 2.1. Collection of Mouse and Human Extraembryonic Tissues

Wild type mouse embryos (CD1) between E8.5 and E18.5 were isolated in ice cold PBS, followed by collection of the amnion, allantois, and visceral yolk sac tissues. The material was washed in ice cold PBS and immediately frozen and stored at −80°C until further processing. BRE : LacZ embryos are transgenic for a gene composed of a BMP-responsive element (BRE) from the *Id1* promotor that drives *β*-galactosidase synthesis that reports BMP-SMAD activity [5]. These embryos were isolated between E7.0 and E8.0 in ice cold PBS, and further processed for *β*-galactosidase staining. CD1 embryos were collected at E8.5 and E9.5 in ice-cold PBS, fixed in 4% paraformaldehyde in PBS and further processed for *in situ* hybridization (ISH).

First and second trimester human extraembryonic tissues were isolated in ice cold PBS and immediately frozen in RLT buffer (Qiagen) until further processing (LUMC). These tissues were collected from voluntary abortions without medical, fetal or obstetrical complications. Human term amnion was collected following planned cesarean sections at the Obstetrics and Gynaecology division, UZ Leuven. Collection of mouse and human tissues was approved by the ethical commission from the KU Leuven (097/2008) and by the Medical Ethical Committee of the Leiden University Medical Center (P08.087), respectively.

### 2.2. *β*-galactosidase Activity and *In Situ* Hybridization

After brief glutaraldehyde/formaldehyde fixation, BRE : LacZ hete-rozygous embryos were washed in PBS and stained for *β*-galactosidase overnight at 30°C in a staining solution of 5-bromo-4-chloro-3-indolyl-beta-D-galacto-pyranoside (X-gal, Fermentas, R0941), as described before [5]. Stained and postfixed embryos were subsequently washed, dehydrated, paraffin embedded and sectioned at 6 *μ*m. Slides were counterstained with Mayer's Hematoxylin.

For *in situ *hybridization, embryos were fixed overnight in 4% paraformaldehyde in PBS at 4°C, washed with PBS and saline, dehydrated and embedded in paraffin, and sectioned at 6 *μ*m. *In situ *hybridization on sections with DIG-labeled antisense riboprobes against *Tbx2 *[20]*, Tbx5 *[21]*, Msx1 *[22]*, Msx2* [23], and *Postn* [24] was performed using an automated platform (Ventana Discovery, Ventana Medical Systems).

### 2.3. Gene Expression Analysis

RNA was extracted and purified with RNeasy purification columns (Qiagen, RNeasy Mini or Micro kit, 74104 and 74004). Reverse transcription was performed using SuperScript III reverse transcriptase (Life Technologies), oligo-dT and random primers (Life Technologies). Real-time qPCR was performed on LightCycler 480 Real-Time PCR System using LightCycler 480 SYBR Green I Master mix (Roche, 4707516001), and all reactions were in technical duplicates. Primers were designed with the online tool of IDT (http://www.idtdna.com/). *Gapdh* and *Ubc* (mouse) and *GAPDH* and **β*-Actin* (human) were used as reference genes for normalization. Mouse primer sequences are (forward primer first) *Afp: *GATGAAACCTATGCCCCTCC, CAAAAGGCCCGAGAAATCTG; *Ap-2: *CGTTACCCTCCTCACGTCACTAG, TTTCGCACACGTACCCAAAGT; *Hand1: *CGAAAGCAAGCGGAAAAGGGAGTT, TTTAGCTCCAGCGCCCAGACTT; *Hand2: *AGGCCTTCAAGGCGGAGATCAA, CCTGTCCGGCCTTTGGTTTTCTTG; *Msx1: *CACCCTACGCAAGCACAAGA, GCAGCTGAGCTGTGGTGAAA; *Msx2: *GCTGCCCTCAGGCTTCAGT, TGCGCCGTATATGGATGCT; *Periostin: *AAGGAAAAGGGTCATACACGTACTTC*, *CCTCTGCGAATGTCAGAATCC**; *Tbx2: *ATCGACAACAACCCCTTTGC, GAGAGTGGGCAGCGTTAGCT; *Tbx4: *TCAACACCTTCCCAACTCAG, GGGAGAACGGAAATAGTGATCG; *Tbx5: *CATCAGTATCACTCGGTACACG, GTTACAACGGGCGATCTAGAG; **ζ*-globin: *GCTTCAAGATCATGACCGCCGT, CGGTGGAGGCTTAGCGGTACTT; *Gapdh: *AAGAAGGTGGTGAAGCAGGC, GCCTCTCTTGCTCAGTGTCC; *Ubc: *TAAAAAGAGCCCTCCTTGTGCT, AGACACCTCCCCATCACAC. Human primer sequences are *PERIOSTIN: *GAAAGGGAGTAAGCAAGGGAG, ATAATGTCCAGTCTCCAGGTTG; *GAPDH: *TGCACCACCAACTGCTTAGC, GGCATGGACTGTGGTCATGAG; **β*-ACTIN: *CACCTTCTACAATGAGCTGCGTGTG, ATAGCACAGCCTGGATAGCAACGTAC. Periostin primers were designed to amplify all known 3 mouse and 4 human transcripts, respectively. Data analysis was performed with qBASEplus software (Biogazelle). Quantification was performed using the “qBASE method”, based on the ΔΔCt method. Two technical replicates were used for each sample and target; standard deviation was calculated from the Ct values of the duplicates and was represented by error bars. Relative RNA levels were obtained by setting the sample with lowest expression (for each target) to 1. The expression of different targets cannot be directly compared. At least five individual samples of E8.5 and E9.5 embryos and at least two individual samples of E13.5 and E18.5 embryos were used in mouse qPCR experiments. For human, one or two individual samples of each mentioned gestational age in first and second trimester were analyzed and 5 individual samples of 38 weeks (term) amnion.

## 3. Results

Immediately after amnion formation the mouse amnion separates and demarcates the extraembryonic region from the embryonic region of the conceptus. At that stage the amnion does not surround the developing embryo yet (Figure 1(a)). At the early organogenesis stage the embryo undergoes an axial rotation and hence it gets enwrapped by its amnion and yolk sac (Figures 1(b)–1(c)). Mouse amniotic membrane is composed of amniotic ectoderm in continuity with the embryonic ectoderm and of amniotic mesoderm sharing its borders with the mesothelium that delineates the visceral yolk sac and the allantois/umbilical cord in the most posterior part of the amnion (Figure 1(a)).

To investigate whether BMP elicits SMAD-mediated BMP signaling around and beyond the stage of amnion closure, we monitored BMP-SMAD activation in amnion of BRE : LacZ reporter embryos. These mouse embryos report the activation of expression of target genes for activated BMP-SMADS [5] (Figures 1(d)–1(h)). Before amnion closure, active BMP-SMAD signaling is most predominant in the amniotic ectoderm component of the amniochorionic fold (Figure 1(d)). After closure high BMP-SMAD signaling levels persist in both layers of the amnion, and the signaling domain starts to expand into the extraembryonic-embryonic junctional region, more specifically in anterior embryonic ectoderm and mesoderm and the most posterior embryonic mesoderm and extraembryonic mesoderm of amnion, allantois, and yolk sac (Figures 1(e)–1(h)).

**Figure 2:**
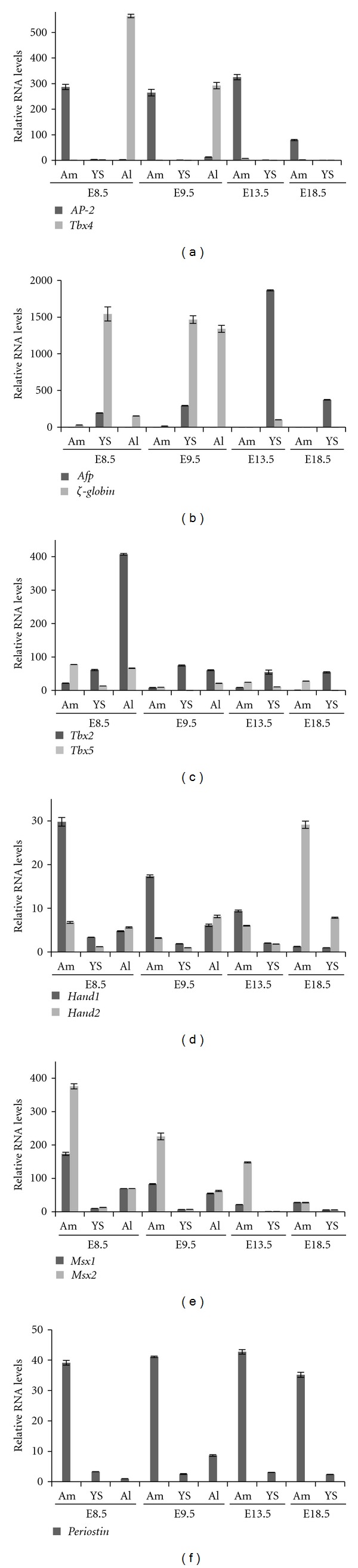
RNA profiling of putative amnion markers in mouse extraembryonic tissues by RT-qPCR. (a)–(b) Validation of microdissected amniotic membrane tissues collected at different stages of development by relative RNA expression analysis of markers for (a) nonneural ectoderm (*Ap-2*) and allantois (*Tbx4*) and (b) primitive red blood cells (*ζ*-*globin*) and yolk sac endoderm (*Afp*). (c)–(e) Expression analysis of acknowledged target genes of SMAD-mediated BMP signaling (*Tbx2*, *Tbx5*, *Hand1*, *Hand2*, *Msx1*, *Msx2*) and (f) *Postn*, in extraembryonic tissues. Relative RNA levels were obtained by setting the sample with lowest expression for each target to 1. The expression of different targets cannot be directly compared. Abbreviations: Am: amnion; Al: allantois; YS: yolk sac.

Amniotic membranes and visceral yolk sacs (called from now on yolk sacs) were microdissected from E8.5, E9.5, E13.5, and E18.5 mouse embryos, covering early organogenesis, organogenesis, and preterm stages of development. In addition, allantoises were isolated from E8.5 and E9.5 embryos. Tissues from at least five embryos were pooled for the E8.5 and E9.5 samples, whilst tissues from older gestational stages were processed individually, and then mRNA was isolated and cDNA synthesized. To assess the quality of these samples, the presence of transcripts enriched in different extraembryonic tissues was evaluated by quantitative reverse transcription-PCR (RT-qPCR). *Tbx4* was used as an allantois-specific marker [25], and *Afp *and **ζ*-globin* markers of the visceral endoderm and primitive haematopoietic cells, respectively, the latter developing in the mesoderm of the visceral yolk sac [26, 27]. An appropriate amnion-specific marker was lacking at this stage but *Ap-2* (*Tfap2A*) was included in the analysis, as we previously reported that this marker for nonneural surface ectoderm and neural crest cells [28] is also abundantly expressed in amniotic ectoderm [13]. This analysis showed that microdissected amniotic membrane samples were not contaminated with allantois (Figure 2(a)) or yolk sac (Figure 2(b)) tissue.

Identically the same amnion, visceral yolk sac, and allantois samples were profiled by RT-qPCR for the expression of 7 selected BMP targets (*Tbx2, Tbx5, Hand1, Hand2, Msx1, Msx2* and *Postn*) as a first selection criterion for identification of (a) putative amnion marker(s).

The transcript levels of the T-box transcription factor encoding gene *Tbx2* are high in allantois and yolk sac when compared to the amnion (Figure 2(c)). The expression in the allantois seemed most predominant at E8.5 and correlates with the expression domain that has been reported previously by *in situ* hybridization [29]. This differential enrichment in the allantois becomes-unlike the *Tbx4 *mRNA expression-progressively lost from E9.5 onwards.The expression domain of *Tbx5* has been documented by *in situ* hybridization in the allantois of E7.5 embryos [30]. The *Tbx5 *profiling does not result in a stable and robust pattern of expression in the different extraembryonic tissues (Figure 2(c)). Hence, we consider neither *Tbx5* nor *Tbx2 *as candidate markers for amniotic membrane.

*Hand1* and* Hand2* encode basic helix-loop-helix transcription factors that are essential for heart and extraembryonic development [31], with *Hand1* seemingly as the favorable candidate marker for amnion (Figure 2(d)). However, in addition to robust expression in the amnion [32] high expression of *Hand1 *has been demonstrated in the yolk sac of a *Hand1 : LACZ*-reporter mouse strain [31]. Moreover, Hand1-deficient embryos show defects in yolk sac vasculature, suggesting that Hand1 is important for yolk sac development [33].

*Msx1* and *Msx2* are Msh homeobox-containing transcription factors involved in neural tube, heart, tooth, limb, and craniofacial development, and are reported to be immediate effectors of BMP signaling (reviewed in [34, 35]). *Msx1* expression has been reported for chick amnion [36], and *Msx2* mRNA was detected in human placenta [37]. This made us investigate the expression of these two genes in mouse extraembryonic tissues. RT-qPCR results showed high expression of both transcripts in amnion early during gestation but also in allantois (Figure 2(e)).

**Figure 3:**
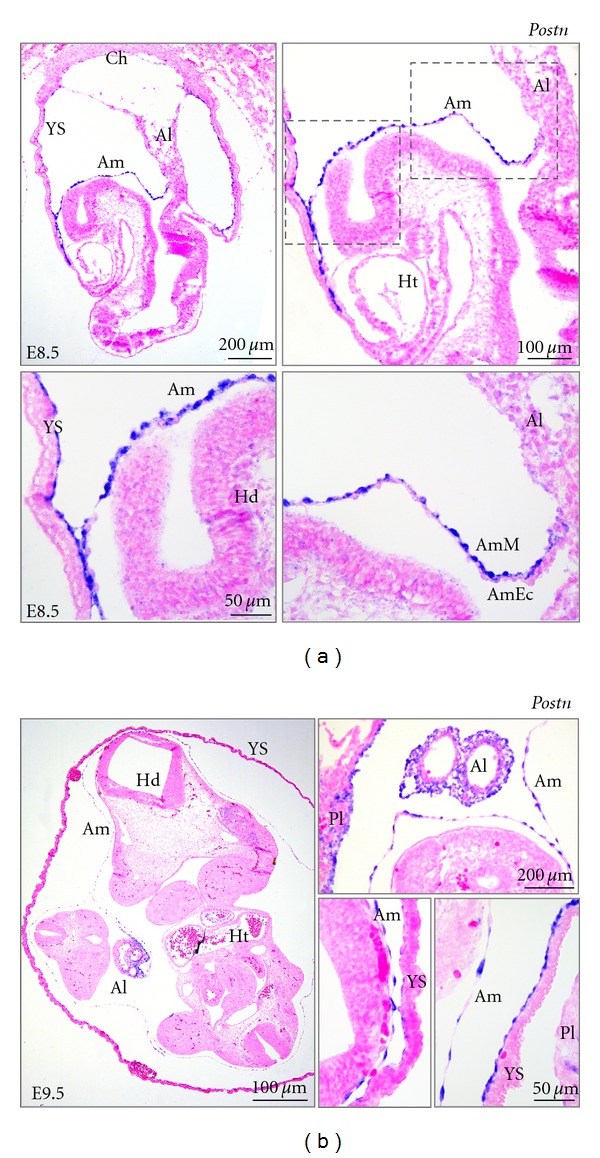
*In situ* hybridization on sections of mouse embryos. (a) RNA localization (blue) of *Postn* in E8.5 mouse embryos. Boxed areas are shown at a higher magnification. *Postn* appears localized in the amniotic mesoderm. (b) RNA localization (blue) of *Postn* in E9.5 mouse embryos. Abbreviations: Am: amnion; Al: allantois; AmM: amniotic mesoderm; AmEC: amniotic ectoderm; Ch: chorionic plate; Ht: heart; Hd: head; Pl: placenta; YS: yolk sac.

*Postn* encodes a poorly described extracellular matrix (ECM) protein. Its expression has been reported in heart and also in the amnion of developing embryos [14, 38], albeit its expression in the other extraembryonic tissues has not been documented. In independent experiments, *Postn* expression was consistently found to be enriched in amnion throughout mouse development (Figure 2(f)), but it was also detected in the yolk sac and allantois. Indeed, *in situ* hybridization analysis confirmed the high expression levels of *Postn *in mouse amnion at E8.5 and E9.5 and highlighted that expression is predominant in amniotic mesoderm (Figures 3(a)–3(b)). *Postn* expression was not detected in the allantois at E8.5, but expression was clearly detected in allantois/umbilical cord at E9.5. Likewise, *Postn* expression was only detected sporadically in mesothelium cells of the visceral yolk sac at E8.5, but *Postn* expression was observed throughout the mesothelium of amnion, allantois, and yolk sac at E9.5 (Figures 3(a)-3(b)).

**Figure 4:**
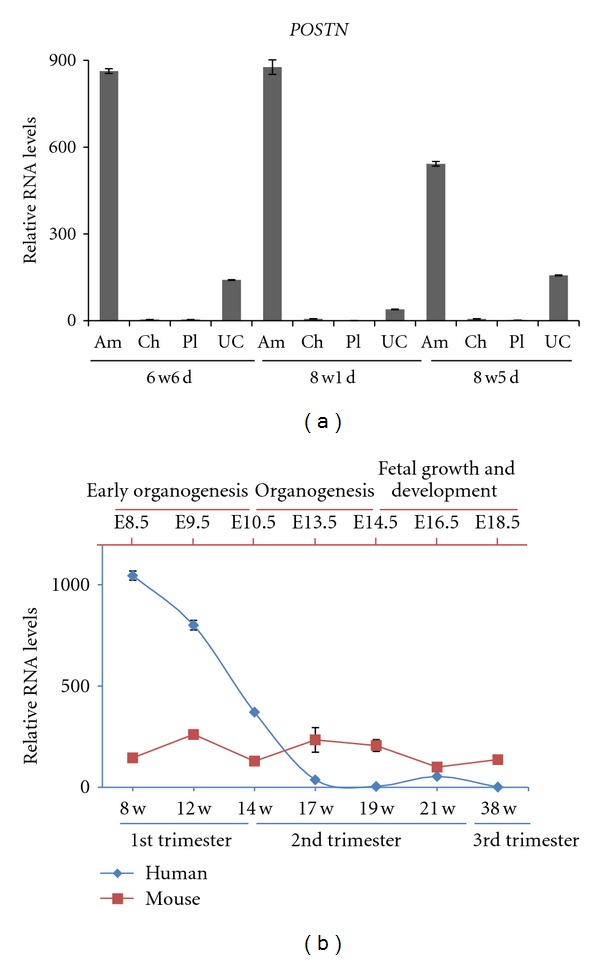
(a) RNA profiling of *POSTN* in human extraembryonic tissues of three individual embryos by RT-qPCR. Gestational age is represented by weeks (w) and days (d). (b) Relative RNA expression of *Postn/POSTN* in mouse and human amnion samples from different developmental stages. In human amnion *POSTN* is expressed at very high levels during the first trimester, followed by a significant drop in expression, whilst* Postn* expression in mouse amnion is stable during gestation. Mouse and human developmental stages are positioned along an arbitrary interval scale. Mouse and human gestation approximates 19.5 days and 38 weeks respectively. Mouse and human developmental stages do not match pairwise. Abbreviations: Am: amnion; Ch: chorion, E: embryonic day; Pl: placenta, UC: umbilical cord, 8–38 w: weeks of gestation.

In human, RT-qPCR analysis of three individual embryos demonstrated that *POSTN *gene expression is highly enriched in amnion during the first trimester of gestation, in comparison with the other extraembryonic tissues*: *chorion, placenta and umbilical cord (Figure 4(a)). *POSTN* expression in the umbilical cord was relatively high as well, similarly to the results for E9.5 mouse allantois (Figure 2(f)). Thus, *POSTN* may be considered as amnion marker in humans too, but it needs to be specified that its expression was only validated by RT-qPCR in isolated extraembryonic tissues (Figure 4(a)). Unlike in mouse, *POSTN *levels in human amnion decrease progressively during gestation (Figure 4(b)).

**Figure 5:**
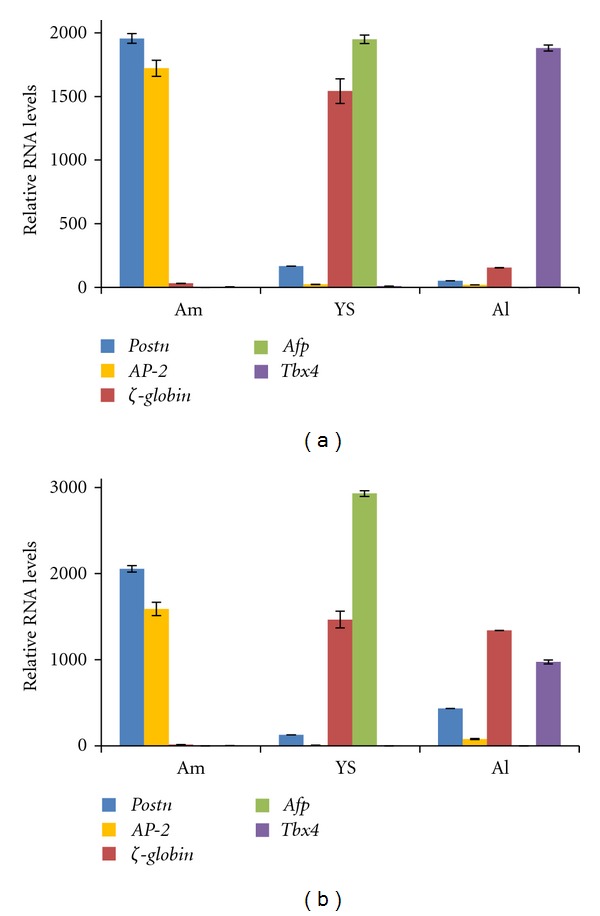
Relative RNA expression of *Postn, AP-2, *ζ*-globin, Afp,* and *Tbx4* in extraembryonic tissues of (a) E8.5 and (b) E9.5 mouse embryos. Here, the relative RNA expression values were multiplied or divided by a scale factor, in order to represent values for different transcripts with different expression levels in a single graph. Expression levels of the different genes cannot be compared. Amnion is enriched in *Postn *and *AP-2*, while visceral yolk sac (*Afp*), allantois (*Tbx4),* and primitive red blood cell (**ζ*-globin*) markers are neglectable. In contrast, yolk sac and allantois express low levels of *Postn *and* AP-2. *Abbreviations: Am: amnion; Al: allantois; YS: yolk sac.

*Postn *expression is enriched in the amniotic membrane, but its expression in extraembryonic tissues is not exclusive to the amnion. To use *Postn *with confidence as an amnion marker we have analyzed a combination of markers for different extraembryonic tissues on extraembryonic samples of E8.5 and E9.5 mouse embryos (Figures 5(a)-5(b)). Based on these results, we conclude that the amniotic membrane is a tissue that expresses relatively high levels of *Postn* and *Ap-2*, and ignorable levels of **ζ*-globin*, *Afp* and *Tbx4* (Figures 5(a)-5(b)).

## 4. Discussion

Given that *Bmp4* expression is patent in developing mouse amnion [16], that genetic mouse models deficient in several components of the BMP signaling cascade develop early amnion defects [6], and that active BMP-SMAD signaling is ongoing in amnion during early organogenesis development, we hypothesized that target genes for BMP are putative amniotic membrane markers. Based on this information, we followed an educated guess approach and identified *Postn* from a preset selection of 7 acknowledged BMP target genes as an amnion-enriched marker gene throughout gestation.

All selected BMP target genes were clearly expressed in mouse amniotic membrane, whereas expression of another BMP target gene, the allantois-specific marker *Tbx4* [25], was indeed not detectable in amnion. The spatial-temporal expression pattern of the different selected BMP target genes followed different trends in the different extraembryonic tissues during development which suggests that they do not all belong to one synexpression group. For instance, *Postn* transcript levels were constantly high in amnion samples throughout gestation, whereas the *Hand1* and the *Msx2* transcripts appeared enriched in the amnion early during organogenesis, but this became less prominent in function of time. The spatial-temporal regionalization of the expression patterns of the respective BMP target genes suggests that the extraembryonic tissues under investigation are exposed to dynamic levels of signaling by (different) BMP morphogens and/or a different regionalization of transcriptional coactivators and repressors may result in a different transcriptional response.

Postn is a secreted ECM protein that has been related to bone and heart development as well as to cancer [19]. The protein is associated with areas of fibrosis; it can directly interact with other ECM proteins, such as fibronectin, tenascin-C, collagen I, collagen V and heparin sulfate proteoglycans. Periostin serves as a ligand for specific integrins, such as *α*v*β*3, *α*v*β*5 and *α*4*β*6 but interacts also with focal adhesion kinases and can thus affect the ability of cells to migrate [19, 39]. *Postn* is expressed in fibroblasts or in cells that adopt fibroblast-like characteristics following an injury event [19]. The extracellular and secreted nature of the Postn protein makes this a less appropriate amniotic membrane marker for FACS-based sorting of cells. Nonetheless, *Postn *is an interesting BMP target gene in the context of the highly stretched amniotic membrane. The expression of *Postn* has been reported to be induced by BMPs, but also by TGF*β*1, and by mechanical stretch [40]. Secreted growth factors of the TGF*β* and BMP families are well known for their involvement in endocardial cushion development within the embryonic heart tube. *Postn* mRNA has been shown to be expressed in the developing mouse embryonic and fetal heart and localizes to the endocardial cushions suggestive of a role in valvulogenesis and valvular disease [41]. Indeed, loss of *Postn* results in the inappropriate differentiation of mesenchymal cushion cells and valvular abnormalities via a TGF*β*-dependent pathway during establishment of the mature heart [19, 40, 42, 43]. So far no amnion defects have been reported in *Postn null *mice. It has, however, been demonstrated recently that Postn interacts with BMP-1. This interaction probably results in enhanced deposition of BMP-1 and BMP-1-mediated proteolytic activation of lysil oxidase on the extracellular matrix, which promotes collagen cross-linking [44]. BMP-1, despite its misleading name, is not a BMP-related growth factor but a Procollagen C-proteinase. Intriguingly, mice deficient in BMP-1 do develop an amnion defect [7].

In the last few years, ECM has been recognized as an important source of regulatory signals in normal tissues and tumors (reviewed in [45]). Recent studies indicate a link between cancer stem cells and their metastatic niches. Together with another ECM protein-tenascin C–*Postn* plays a key role as metastasis niche component for breast-derived tumour-initiating cells that invade the lungs [46]. By enhancing Wnt and Notch signaling in cancer cells, Postn and tenascin C provide physical and signaling support for metastasis-initiating cells. *Postn* deficient mice develop mammary tumors, but their ability to metastasize to the lungs is significantly diminished compared to tumors in wild-type mice. Malanchi et al. propose that the role of Postn in progression of lung metastasis is to concentrate Wnt ligands in the metastatic niche for the stimulation of stem-like metastasis-initiating cells [46]. Postn promotes tumor metastasis and facilitates invasion in the tumor microenvironment also in colorectal, pancreatic, oral, prostate, esophageal, and ovarian cancer [47–52]. Perhaps the presence of Postn in amnion may contribute to the reprogramming capacities of amniotic membrane cells in cell culture.

*Postn* is enriched in mouse amnion, and its expression level remains fairly constant during gestation. *Postn* mRNA appeared localized in the amniotic mesoderm, while the amniotic ectoderm appeared negative (Figure 3(a)). This was especially clear before embryo turning at E8.5 when amnion is less stretched. *POSTN *can be considered a suitable amnion marker in humans too, although its transcript levels decrease progressively during gestation. The timing of the observed decrease seems to correlate with the phase when amnion and chorion physically fuse, and it is therefore tempting to speculate that changes in mechanical stretch and pressure/contact with the chorion as compared to the previously surrounding fluid attenuates the *POSTN *levels in the amniotic membrane. During the first trimester of gestation, *POSTN* is highly enriched in human amnion comparing to the other extraembryonic tissues (chorion, placenta and umbilical cord), but it is not restricted to amnion only, as demonstrated by its expression in the umbilical cord. The expression of *POSTN* throughout gestation may be dynamic in different extraembryonic tissues because *POSTN* mRNA has been detected by *in situ* hybridization in the stromal cells of human term placenta [53]. In this study the authors do not report *POSTN* expression levels in other nonplacental extraembryonic tissues. In any case, analyzing sets of markers for human amniotic membrane instead of using a single marker gene is preferable, and more research needs to be performed in this direction.

Our *in situ* hybridization analysis confirms that mouse *Postn *is enriched in amnion during early organogenesis and highlights furthermore that it is expressed in amniotic mesoderm. However, *Postn* expression can be observed in mesothelium of the visceral yolk sac and allantois as well. To bypass making premature conclusions on amnion identity based on analysis of *Postn* expression levels only, we suggest to categorize amnion, visceral yolk sac and allantois by expression profiling of a set of different marker genes. Mouse amniotic membrane is characterized during early organogenesis by high levels of *Postn* and *Ap-2*, low levels of **ζ*-globin* expression, and ignorable levels of *Afp* and *Tbx4* (Figure 5). Visceral yolk sac can be classified as the tissue with high expression levels of *Afp *and **ζ*-globin,* with moderate expression of *Postn* and *Ap-2*, and absence of *Tbx4*; whereas the allantois is characterized by high *Tbx4 *expression level and weak expression of *Postn, Ap-2* and **ζ*-globin. Afp* expression was undetectable in the allantois (Figures 2(b) and 5). Since **ζ*-globin* is a marker of primitive haematopietic cells in the yolk sac, it is not advisable to use this marker beyond early organogenesis development.

In summary, we propose using *Postn* and *Ap-2 *as a marker set enriched in mouse amniotic membrane and we propose a combination of markers as transcriptional signature for the different mouse extraembryonic tissues. The unbiased identification of additional markers, preferentially of amnion-enriched intracellular proteins and/or membrane proteins, and the use of panels of amnion markers should be further encouraged.

## References

[1] Parolini O, Alviano F, Bagnara GP (2008). Concise review: isolation and characterization of cells from human term placenta: outcome of the First International Workshop on Placenta Derived Stem Cells. *Stem Cells*.

[2] Dobreva MP, Pereira PNG, Deprest J, Zwijsen A (2010). On the origin of amniotic stem cells: of mice and men. *International Journal of Developmental Biology*.

[3] Marcus AJ, Coyne TM, Rauch J, Woodbury D, Black IB (2008). Isolation, characterization, and differentiation of stem cells derived from the rat amniotic membrane. *Differentiation*.

[4] Ditadi A, De Coppi P, Picone O (2009). Human and murine amniotic fluid c-Kit+Lin- cells display hematopoietic activity. *Blood*.

[5] Monteiro RM, Chuva de Sousa Lopes SM, Korchynskyi O, ten Dijke P, Mummery CL (2004). Spatio-temporal activation of Smad1 and Smad5 in vivo: monitoring transcriptional activity of Smad proteins. *Journal of Cell Science*.

[6] Pereira PNG, Dobreva MP, Graham L, Huylebroeck D, Lawson KA, Zwijsen A (2011). Amnion formation in the mouse embryo: the single amniochorionic fold model. *BMC Developmental Biology*.

[7] Suzuki N, Labosky PA, Furuta Y (1996). Failure of ventral body wall closure in mouse embryos lacking a procollagen C-proteinase encoded by Bmp1, a mammalian gene related to Drosophila tolloid. *Development*.

[8] Gersdorff N, Müller M, Otto S, Poschadel R, Hübner S, Miosge N (2005). Basement membrane composition in the early mouse embryo day 7. *Developmental Dynamics*.

[9] Inman KE, Downs KM (2007). The murine allantois: emerging paradigms in development of the mammalian umbilical cord and its relation to the fetus. *Genesis*.

[10] Cross JC (2005). How to make a placenta: mechanisms of trophoblast cell differentiation in mice—a review. *Placenta*.

[11] Wang X, Bornslaeger EA, Haub O (1996). A candidate gene for the amnionless gastrulation stage mouse mutation encodes a TRAF-related protein. *Developmental Biology*.

[12] Zhang H, Bradley A (1996). Mice deficient for BMP2 are nonviable and have defects in amnion/chorion and cardiac development. *Development*.

[13] Chang H, Huylebroeck D, Verschueren K, Guo Q, Matzuk MM, Zwijsen A (1999). Smad5 knockout mice die at mid-gestation due to multiple embryonic and extraembryonic defects. *Development*.

[14] Bosman EA, Lawson KA, Debruyn J (2006). Smad5 determines murine amnion fate through the control of bone morphogenetic protein expression and signalling levels. *Development*.

[15] Pereira PNG, Dobreva MP, Cornelis F

[16] Lawson KA, Dunn NR, Roelen BAJ (1999). Bmp4 is required for the generation of primordial germ cells in the mouse embryo. *Genes and Development*.

[17] Ishimura A, Chida S, Osada SI (2008). Man1, an inner nuclear membrane protein, regulates left-right axis formation by controlling nodal signaling in a node-independent manner. *Developmental Dynamics*.

[18] Madabhushi M, Lacy E (2011). Anterior visceral endoderm directs ventral morphogenesis and placement of head and heart via BMP2 expression. *Developmental Cell*.

[19] Conway SJ, Doetschman T, Azhar M (2011). The inter-relationship of periostin, TGF*β*, and BMP in heart valve development and valvular heart diseases. *TheScientificWorldJournal*.

[20] Shirai M, Imanaka-Yoshida K, Schneider MD, Schwartz RJ, Morisaki T (2009). T-box 2, a mediator of Bmp-Smad signaling, induced hyaluronan synthase 2 and Tgf*β*2 expression and endocardial cushion formation. *Proceedings of the National Academy of Sciences of the United States of America*.

[21] Bruneau BG, Logan M, Davis N (1999). Chamber-specific cardiac expression of Tbx5 and heart defects in Holt- Oram syndrome. *Developmental Biology*.

[22] Hill RE, Jones PF, Rees AR (1989). A new family of mouse homeo box-containing genes: molecular structure, chromosomal location, and developmental expression of Hox-7.1. *Genes & Development*.

[23] Mackenzie A, Ferguson MWJ, Sharpe PT (1992). Expression patterns of the homeobox gene, Hox-8, in the mouse embryo suggest a role in specifying tooth initiation and shape. *Development*.

[24] Délot EC, Bahamonde ME, Zhao M, Lyons KM (2003). BMP signaling is required for septation of the outflow tract of the mammalian heart. *Development*.

[25] Naiche LA, Arora R, Kania A, Lewandoski M, Papaioannou VE (2011). Identity and fate of Tbx4-expressing cells reveal developmental cell fate decisions in the allantois, limb, and external genitalia. *Developmental Dynamics*.

[26] Dziadek MA, Andrews GK (1983). Tissue specificity of alpha-fetoprotein messenger RNA expression during mouse embryogenesis. *The EMBO Journal*.

[27] Barker JE (1968). Development of the mouse hematopoietic system. I. Types of hemoglobin produced in embryonic yolk sac and liver. *Developmental Biology*.

[28] Mitchell PJ, Timmons PM, Hebert JM, Rigby PWJ, Tjian R (1991). Transcription factor AP-2 is expressed in neural crest cell lineages during mouse embryogenesis. *Genes and Development*.

[29] Harrelson Z, Kelly RG, Goldin SN (2004). Tbx2 is essential for patterning the atrioventricular canal and for morphogenensis of the outflow tract during heart development. *Development*.

[30] Chapman DL, Garvey N, Hancock S (1996). Expression of the T-box family genes, *Tbx1-Tbx5*, during early mouse development. *Developmental Dynamics*.

[31] Morikawa Y, Cserjesi P (2004). Extra-embryonic vasculature development is regulated is regulated by the transcription factor HAND1. *Development*.

[32] Tanaka M, Chen Z, Bartunkova S, Yamasaki N, Izumo S (1999). The cardiac homeobox gene Csx/Nkx2.5 lies genetically upstream of multiple genes essential for heart development. *Development*.

[33] Firulli AB, McFadden DG, Lin Q, Srivastava D, Olson EN (1998). Heart and extra-embryonic mesodermal defects in mouse embryos lacking the bHLH transcription factor Hand1. *Nature Genetics*.

[34] Ramos C, Robert B (2005). msh/Msx gene family in neural development. *Trends in Genetics*.

[35] Suzuki A, Ueno N, Hemmati-Brivanlou A (1997). Xenopus msx1 mediates epidermal induction and neural inhibition by BMP4. *Development*.

[36] Fliniaux I, Viallet JP, Dhouailly D (2004). Signaling dynamics of feather tract formation from the chick somatopleure. *Development*.

[37] Quinn LM, Latham SE, Kalionis B (2000). The homeobox genes MSX2 and MOX2 are candidates for regulating epithelial-mesenchymal cell interactions in the human placenta. *Placenta*.

[38] Rios H, Koushik SV, Wang H (2005). periostin null mice exhibit dwarfism, incisor enamel defects, and an early-onset periodontal disease-like phenotype. *Molecular and Cellular Biology*.

[39] Horiuchi K, Amizuka N, Takeshita S (1999). Identification and characterization of a novel protein, periostin, with restricted expression to periosteum and periodontal ligament and increased expression by transforming growth factor *β*. *Journal of Bone and Mineral Research*.

[40] Wang Z, Ouyang G (2012). Periostin: a bridge between cancer stem cells and their metastatic niche. *Cell Stem Cell*.

[41] Li G, Jin R, Norris RA (2010). Periostin mediates vascular smooth muscle cell migration through the integrins *ανβ*3 and *ανβ*5 and focal adhesion kinase (FAK) pathway. *Atherosclerosis*.

[42] Snider P, Hinton RB, Moreno-Rodriguez RA (2008). Periostin is required for maturation and extracellular matrix stabilization of noncardiomyocyte lineages of the heart. *Circulation Research*.

[43] Kruzynska-Frejtag A, Machnicki M, Rogers R, Markwald RR, Conway SJ (2001). Periostin (an osteoblast-specific factor) is expressed within the embryonic mouse heart during valve formation. *Mechanisms of Development*.

[44] Oka T, Xu J, Kaiser RA (2007). Genetic manipulation of periostin expression reveals a role in cardiac hypertrophy and ventricular remodeling. *Circulation Research*.

[45] Oskarsson T, Massagué J (2012). Extracellular matrix players in metastatic niches. *The EMBO Journal*.

[46] Malanchi I, Santamaria-Martínez A, Susanto E (2012). Interactions between cancer stem cells and their niche govern metastatic colonization. *Nature*.

[47] Bao S, Ouyang G, Bai X (2004). Periostin potently promotes metastatic growth of colon cancer by augmenting cell survival via the Akt/PKB pathway. *Cancer Cell*.

[48] Baril P, Gangeswaran R, Mahon PC (2007). Periostin promotes invasiveness and resistance of pancreatic cancer cells to hypoxia-induced cell death: role of the *β*4 integrin and the PI3k pathway. *Oncogene*.

[49] Siriwardena BSMS, Kudo Y, Ogawa I (2006). Periostin is frequently overexpressed and enhances invasion and angiogenesis in oral cancer. *British Journal of Cancer*.

[50] Tischler V, Fritzsche FR, Wild PJ (2010). Periostin is up-regulated in high grade and high stage prostate cancer. *BMC Cancer*.

[51] Michaylira CZ, Wong GS, Miller CG (2010). Periostin, a cell adhesion molecule, facilitates invasion in the tumor microenvironment and annotates a novel tumor-invasive signature in esophageal cancer. *Cancer Research*.

[52] Zhu M, Saxton RE, Ramos L (2011). Neutralizing monoclonal antibody to periostin inhibits ovarian tumor growth and metastasis. *Molecular Cancer Therapeutics*.

[53] Sasaki H, Roberts J, Lykins D, Fujii Y, Auclair D, Chen LB (2002). Novel chemiluminescence assay for serum periostin levels in women with preeclampsia and in normotensive pregnant women. *American Journal of Obstetrics and Gynecology*.

